# Continous application of bioorganic fertilizer induced resilient culturable bacteria community associated with banana Fusarium wilt suppression

**DOI:** 10.1038/srep27731

**Published:** 2016-06-16

**Authors:** Lin Fu, Yunze Ruan, Chengyuan Tao, Rong Li, Qirong Shen

**Affiliations:** 1 Jiangsu Key Lab for Organic Solid Waste Utilization, National Engineering Research Center for Organic-based Fertilizers, Jiangsu Collaborative Innovation Center for Solid Organic Waste Resource Utilization, Nanjing Agricultural University, Nanjing, China; 2 Hainan key Laboratory for Sustainable Utilization of Tropical Bio-resources, College of Agriculture, Hainan University, Haikou, China

## Abstract

Fusarium wilt of banana always drives farmers to find new land for banana cultivation due to the comeback of the disease after a few cropping years. A novel idea for solving this problem is the continuous application of bioorganic fertilizer (BIO), which should be practiced from the beginning of banana planting. In this study, BIO was applied in newly reclaimed fields to pre-control banana Fusarium wilt and the culturable rhizobacteria community were evaluated using Biolog Ecoplates and culture-dependent denaturing gradient gel electrophoresis (CD-DGGE). The results showed that BIO application significantly reduced disease incidences and increased crop yields, respectivly. And the stabilized general bacterial metabolic potential, especially for the utilization of carbohydrates, carboxylic acids and phenolic compounds, was induced by BIO application. DGGE profiles demonstrated that resilient community structure of culturable rhizobacteria with higher richness and diversity were observed in BIO treated soils. Morever, enriched culturable bacteria affiliated with *Firmicutes*, *Gammaproteobacteria* and *Actinobacteria* were also detected. In total, continuous application of BIO effectively suppressed Fusarium wilt disease by stabilizing culturable bacterial metabolic potential and community structure. This study revealed a new method to control Fusarium wilt of banana for long term banana cultivation.

Fusarium wilt of bananas (*Musa* spp.), which is also known as Panama disease, is caused by the soil-borne fungus *Fusarium oxysporum* f. sp. *cubense* (Foc). It is one of the most serious and destructive plant diseases, causing economical losses to banana crop and becoming the primary constraint in banana production at the global level^1^. In recent decades, a new variant of Foc called Tropical Race 4 (Foc-TR4), which was first detected in Asia in 1990s, has a particularly devastating effect on the popular Cavendish cultivar and posed a serious threat to the banana industry worldwide^2^. Early studies showed that susceptible varieties could not be successfully replanted^3^, and cultural practices and chemical control were often insufficiently effective^4^,^5^,^6^, resulting in the loss of many banana plantations. Consequently, banana growers often have no choice but to reclaim new areas that are free of disease to grow banana^7^. However, banana Fusarium wilt will also soon appear in the newly reclaimed fields if inappropriate cultivation and poor management of banana orchards are practiced^8^. Therefore, it is urgent and necessary to explore novel ways to grow healthy banana plants to maintain the sustainable development of banana industry.

The difficulty in controlling Fusarium wilt has encouraged relevant research to develop sustainable strategies, and biological control is the most promising alternative to chemical control, given its eco-friendly nature and the discovery of novel mechanisms of plant protection associated with certain microorganisms^9^. Combination of biocontrol microbes and composts to creat bio-organic fertilizers (BIOs) could enhance the activity of functional agents^10^,^11^,^12^,^13^. In a previous study, a novel BIO that integrated *Bacillus amyloliquefaciens* NJN-6 with matured compost was able to efficiently control banana Fusarium wilt disease in pot experiments^14^ and in field experiments in a banana orchard that had been continuously cropped with bananas for 12 years with serious Fusarium wilt disease^15^. However, the suitability of BIO applications in maintaining sustainable and effective biocontrol efficacy in the newly reclaimed fields remained largely ignored since most farmers could not recognize the importance of pre-control of this soil-borne disease from the beginning of the cultivation. Based on the present study, the potential mechanisms invovled in disease suppression of BIO mainly correlated to colonizing the rhizosphere by forming biofilm^12^, the production of anti-fungal compounds^14^ and the induced general suppression of soil microbial community^15^. Thus, in the perspective of cuturable microbial community, the potential mechanisms of disease suppression with BIO may be disclosed in a broader way.

Soil is a complex and dynamic environment in which the biological activity is mostly governed by microorganisms^16^. The rhizosphere, soil adhering to the plant roots, is the place where complex biological and ecological processes occur, such as the production of antibiotics, pathogenesis and its counterparts, the geochemical cycling of minerals, and plant protection^17^. The biocontrol approach relies on the hypothesis that the rhizosphere microbial community can be manipulated to promote the natural disease suppression^18^. The ability to monitor a disease suppressive microbial community in the rhizosphere is presently hindered by the lack of knowledge about rhizosphere microbial ecology and a restricted ability to identify and characterize microbial communities that are associated with healthy and infected roots^19^. Hence, it is required to better understand the structural and functional diversity of microbial communities in the rhizosphere and their succession during plant development in order to explore the efficient application of biological control agents.

In recent years, rapid-developed molecular techniques greatly contributed to our understanding of the microbial diversity in soil^20^. However, the most numerically abundant in terms of genetic material are not equal to the most numerically abundant in cellular activity^21^,^22^. Furthermore, some studies indicate that culturable bacteria are important to soil ecosystem functions and may provide an ecologically relevant complement to culture-independent community characterizations^23^,^24^,^25^. Recently, Biolog Ecoplates have been widely applied in comparative soil analyses and have been shown to be a powerful and sensitive analytical tool for demonstrating differences in soil microbiological characteristics^26^,^27^. Also, the culture-dependent DGGE analysis is universally applicable^23^, and it allows the description of specific culturable bacteria in soil settings^24^ that ideally suited the purpose of this research. Here we described the culturable part of rhizobacterial community based on culture-dependent approaches, in expectation of a deeper understanding of the soil functional and metabolic potential rather than the characteristics of the whole soil microbial community.

In this study, the effects of a novel fertilization mode that applied BIO in newly reclaimed fields, on the biocontrol efficacy, crop yield and rhizosphere culturable bacterial community of banana plants were investigated over four subsequent years (2009–2012) using the combined methods of CD-DGGE and Biolog in Lingao county, Hainan province, China. The objectives of this study were to 1) investigate whether the reclaimed fields amended with BIO from the beginning of banana planting can maintain low disease incidence in subsequent years as compared to the fields non-amended with BIO; 2) investigate the effects of BIO on the culturable rhizobacterial community; and 3) decipher the potential biocontrol mechanism of the BIO on Panama disease in the newly reclaimed fields based on culture-dependent methods.

## Results

### Effects of BIO application on disease incidence of Fusarium wilt and banana yield

**Figure 1:**
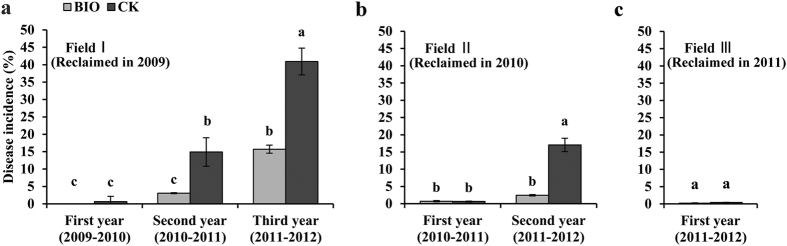
Fusarium wilt disease incidence for BIO and CK treatments in different reclaimed fields corresponding to various cropping years. (**a**) Planting bananas for three years, field reclaimed in 2009. (**b**) Planting bananas for two years, field reclaimed in 2010. (**c**) Planting bananas for one year, field reclaimed in 2011. Bars with different letters indicate significant differences among the treatments, as defined by LSD test (*P* < 0.05).

As shown in Fig. 1, the disease incidence presented a rising trend with the lengthening of planting years regardless of the BIO and CK treatments. There was no significant difference between BIO and CK treatments after one-year of banana planting in different reclaimed fields. However, in the continuous cropping fields (Field Ι and Field Π) after two or three years, the BIO application significantly (*P* < 0.05) reduced the disease incidence in comparison with the control, indicating that BIO application could effectively control the outbreak of Fusarium wilt disease in the newly reclaimed banana fields.

**Figure 2:**
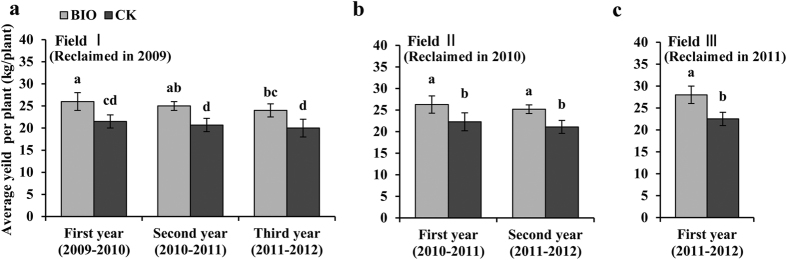
The average yield per banana plant for BIO and CK treatments in different reclaimed fields corresponding to various cropping years. (**a**) Planting bananas for three years, field reclaimed in 2009; (**b**) Planting bananas for two years, field reclaimed in 2010; (**c**) Planting bananas for one year, field reclaimed in 2011. Bars with different letters indicate significant differences among the treatments, as defined by LSD test (*P* < 0.05).

The application of bio-organic fertilizer significantly enhanced the banana yield per plant by 20.9% (Field Ι), 17.9% (Field Π), and 24.4% (Field ΙΙΙ) for the first year; 20.8% (field Field Ι) and 19.4% (Field Π) for the second year; and 20.0% (Field Ι) for the third year as compared to corresponding CK treatments (Fig. 2).

### Effects of BIO application on the metabolic activity of culturable rhizobacteria

#### The general bacterial metabolic activity for carbon substrates utilization

**Figure 3:**
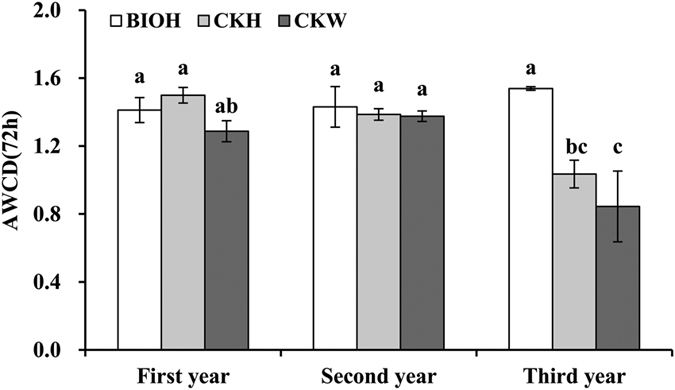
Average well color development (AWCD) of metabolized substrates in Biolog Ecoplate of rhizosphere soil samples amened with different fertilizers. The data shown here are the means of 31 substrate well absorbance values at 72 h. Error bars represent the standard deviations (n = 3), and different letters indicate the significant difference (*P* < 0.05). BIO1H, BIO2H, BIO3H refer to the healthy plants in fields amend with BIO for one year, two years and three years; CK1H, CK2H, CK3H and CK1W, CK2W, CK3W refer to the healthy plants and wilt plants in CK treated areas for one year, two years and three years respectively.

Average well color development (AWCD) used as an estimate of general bacterial activity was calculated based on the results of Biolog Ecoplates in 72 h. As shown in Fig. 3, no significant difference in bacterial community metabolic activity between BIO and CK treatments was observed for the first two years. Remarkably, a significant higher use of 31 types of carbon substrates was observed in the BIO treated rhizosphere soil as compared to CK in the third year. The lowest AWCD was observed in the wilted plant rhizosphere soil sample from the control in the third year. Furthermore, the carbon metabolic activity of the soil bacterial communities showed a declining trend in the CK treatment along with the cropping years, and in contrast, the BIO-treated soils presented stable levels in general bacterial metabolic activity .

#### Kinetic profiles for different groups of carbon sources

The substrates in Biolog Ecoplates can be assigned to guilds of carbohydrates, amino acids, carboxylic acids, amines, phenolic acids and polymers according to their chemical nature^28^ (Supplementary Table S2). The time course changes of the six types carbon substrates AWCD data was successfully fitted a modified form of the logistic equation (χ^2^ ≤ 0.013 and R^2^ ≥ 0.97, data not shown) and the parameters were calculated (Table 1). For the first year, no significant difference was observed between BIO and CK in the carrying capacity (k) and the rate of consumption (r) of different groups of carbon sources, although the BIO treatment showed a slightly lower consumption of amino acids, polymers, and amines than the control. However, a significantly higher consumption of all six types of carbon sources in the soils amended with BIO was observed relative to the control in the second and third years (*P* < 0.05). Meanwhile, the consumption ability for different groups of carbon sources in CK treatment showed a declining trend along with the cropping years. Notably, the increasing or stable carrying capacity and consumption rate for carbohydrates, carboxylic acids and phenolic compounds were observed in the BIO-amended soils along with the cropping years.

#### Principal component analysis of the utilization ability of substrate classes at AWCD_0.8_

**Figure 4:**
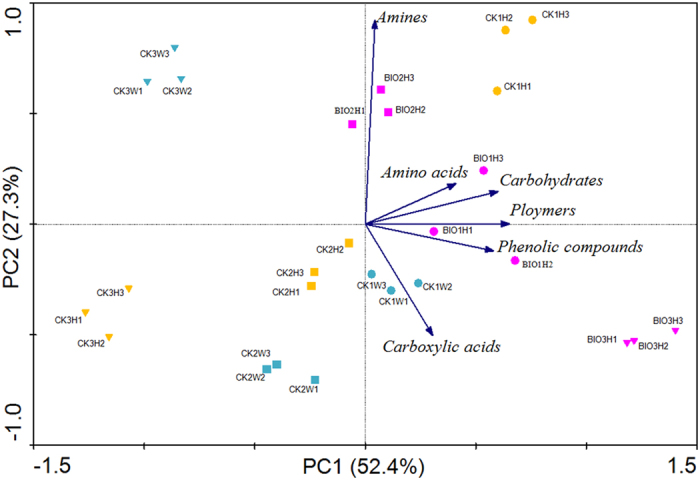
PCA ordination of carbon substrate utilization patterns for different rhizosphere soil samples. The percentage of total variance as explained by each axis is shown. All values are based on AWCD data equivalent to 0.8, the various samples are represented by symbols of different shape or color, and each sample has three replicates. The nany arrowed lines represent the six guilds of carbon sources. BIO1H, BIO2H, BIO3H refer to the healthy plants in fields amend with BIO for one year, two years and three years; CK1H, CK2H, CK3H and CK1W, CK2W, CK3W refer to the healthy plants and wilt plants in CK treated areas for one year, two years and three years respectively.

As shown in Fig. 4, the PCA results revealed that metabolic profiles for the soil samples corresponding to one year banana planting fields were similar to each other (in PC1 dirrection). However, soil samples derived from two and three years banana planting fields were significantly distinguished between BIO and CK treatments (with separation along PC1, left to right). The substrate classes of amino acids, carbohydrates, polymers and phenolic compounds were decisively responsible for the differences among soil samples. In addition, none of the investigated substrate classes was used well in the CK treatment with continuous planting for two and three years. In particular, carboxylic acids and phenolic compounds were metabolized more efficiently in BIO3H rhizosphere soil.

### Effects of BIO application on the genetic diversity of culturable rhizobacteria

**Figure 5:**
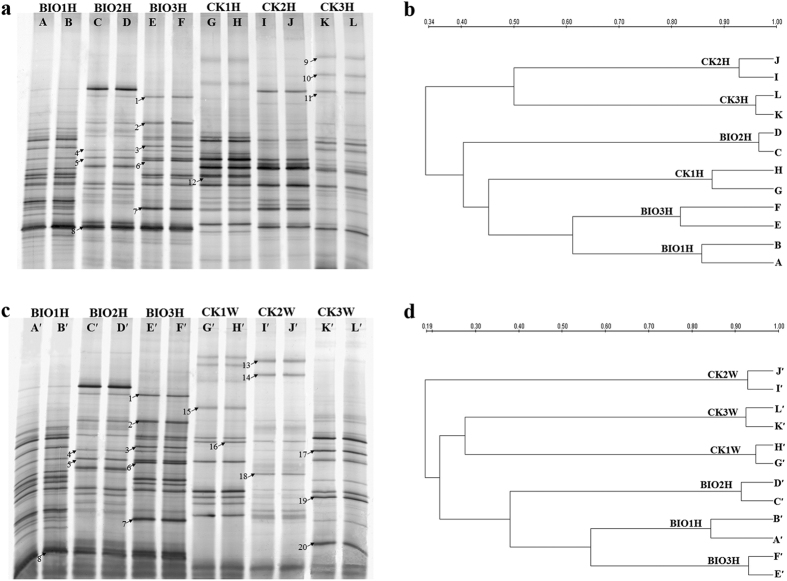
(**a**,**c**) The DGGE fingerprinting of culturable bacteria in different rhizosphere soil samples. (**b**,**d**) The cluster analysis of DGGE profiles based on UPGMA algorithm. Scale bar represents the percent similarity. BIO1H, BIO2H, BIO3H refer to the healthy plants in fields amend with BIO for one year, two years and three years; CK1H, CK2H, CK3H and CK1W, CK2W, CK3W refer to the healthy plants and wilt plants in CK treated areas for one year, two years and three years respectively. The letters A-L are the lanes of the DGGE profile. AB, CD, EF, GH, IJ, and KL are the duplicates of the samples BIO1H, BIO2H, BIO3H, CK1H, CK2H and CK3H, respectively. A′B′, C′D′, E′F′, G′H′, I′J′, and K′L′ are the duplicates of the samples BIO1H, BIO2H, BIO3H, CK1W, CK2W and CK3W, respectively. The bands labeled with arrows were excised, re-amplified and sequenced for bacterial taxonomic assessment.

Changes in the culturable bacteria community structures of the rhizosphere soil were monitored by using PCR-DGGE analysis of the 16S rRNA gene. Because of the limited numbers of loading wells in the polyacrylamide gel, we could not use all the samples together (including replicates of each sample) on one gel at the same time. Therefore, differences among samples were observed in the two gels during the comparison of BIOH samples with the CKH (Fig. 5a) and CKW (Fig. 5c) samples separately. A total of 37 (Fig. 5a) and 36 (Fig. 5c) ribotypes (band positions) were identified for each DGGE profile. The richness (R) and diversity (*H*´) indexes of the BIO treatments were higher in comparison with the CK corresponding to different planting years, indicating that BIO application resulted in higher soil culturable bacterial richness and diversity (Supplementary Table S3). In addition, lowest richness and diversity indexes were observed in soil samples from diseased plants in CK. Moreover, no significant difference in the evenness (E) among all soil samples was found.

The similarity of culturable bacterial community structures was evaluated on the basis of UPGMA analysis (Fig. 5b,d). As shown in Fig. 5b, with respect to the soil samples of healthy plants between BIO and CK treatments, the tree was composed of two primary clusters separated at 34% similarity. The BIO-treated soil samples and CK soil sample from the field with one-year banana planting were within the same cluster, and it was distinct from the soil samples of the second and third year in CK treatment. This finding indicated that the banding patterns of the culturable bacterial communities under CK treatment changed significantly as the cropping years were prolonged. In contrast, the BIO application maintained the structure at a relatively balanced status (the samples were within a cluster in the tree) in the continuous cropping mode. When compared to the soil samples of wilted plants in the CK treatment, the samples of healthy plants amended with BIO were grouped together, and the CK treatment samples split from this group at approximately 19% similarity (Fig. 5d). These results indicated that the culturable bacterial community structures in the rhizosphere soil between healthy and wilted plants were markedly different.

The DGGE bands (marked in Fig. 5a,c) that discriminated among treatments were excised and sequenced (Table 2). Specifically, bands 1, 2, 4, 5, 7, 8, and 12 were affiliated with *Bacillus megaterium*, *Bacillus stratosphericus*, *Rhizobium* sp., *Burkholderia* sp., *Acinetobacter baumannii*, *Brevibacillus formosu*, and *Sinorhizobium meliloti*, respectively (Fig. 5a, Table 2). All of which showed primary or increased dominance in BIO-treated rhizosphere soil with the lengthening of planting years, and those disappeared or became less dominant in the CK treatment of healthy and wilted plants. Band 3 and band 6, which were identified as *Pseudomonas fluorescens* and *Actinobacterium*, respectively, were only detected in the BIO-treated rhizosphere soil in third year (Fig. 5a, Table 2). Bands 9, 10, and 11 were identified as *Chryseobacterium gleum*, *Flavobacterium* sp., and *Chitinophaga* sp., respectively, and appeared in the rhizosphere soil of healthy plants of CK treatment (Fig. 5a, Table 2). Bands 13, 14, 15, 16, 17, 18, 19, and 20 were affiliated with *Agrobacterium tumefaciens*, *Rhizobium huautlense*, *Pseudoxanthomonas* sp., *Chitinophagaceae bacterium*, *Pedobacter* sp., *Cronobacter sakazakii*, *Novosphingobium* sp., and *Sphingobium* sp., respectively, and were highly prevalent in the CK-treated rhizosphere soil of wilted plants, but those were not detected or very weak in rhizosphere soil of healthy plants (Fig. 5c, Table 2).

## Discussion

In this research, a novel healthy fertilization method, which BIO was applied in parallel with banana planting in the newly reclaimed fields, was firstly reported to prevent and suppress Fusarium wilt disease of banana. The results showed that continuous application of BIO significantly reduced the banana Fusarium wilt disease incidence as compared to CK. The results were consistent with previously published studies showing that BIO application resulted in prominent suppression of banana Fusarium wilt in pot expetiments and in disease already happened fields^14^,^15^. Previous research also indicated that bio-organic fertilizer possesses a more suppressive effect on soil-borne disease than compost alone^10^.

The results of the higher mean weights of banana fruits in BIO treatment were similar to those of a comparative study in which bio-formulations with chitin and the PGPR strain (*Pseudomonas fluorescens*) significantly increased the morphological characteristics and yield of banana^11^. This result is also consistent with Rivera-Cruz *et al*.^29^ who reported that the banana shoot and root biomass were significantly increased using either poultry manure or banana waste as the carrier for the bacterial inoculant. Thus, the increase in yield observed in the present study might be attributed to the comprehensive effects of both growth-promotion and biocontrol.

Measurements of functional diversity provide an assessment of ecosystem health^30^. With respect to soil bacterial activity as indicated by the AWCD values in this study, BIO applications had a positive influence on the overall catabolic activity of the culturable rhizobacteria, and the bacterial metabolic activity was depressed with the continuous cropping in soils amended with pig manure (CK). Similar to our study, Perez-Piqueres *et al*.^31^ reported that soil biological modifications depended on the soil and organic matter applied. They further found that addition of different organic amendments to the same soil caused different effects, with some enhancing the metabolic activity and some not. Composts are often employed as an important source of nutrients to be used by microorganisms to improve soil quality, notably by contributing to general suppression through enhanced soil microbial biomass and activity^32^. Hence, when compared to the depressed bacterial activity in the CK treatment, the stable soil bacterial activities applied with BIO might be related to the general suppression of soil to Foc, because an active microbial community was thought to be more efficient at controlling soil pathogens.

Regarding the kinetic analysis of biolog datasets, color development curves have been successfully fitted into the modified logistic equation, which provides terms describing the lag (s), maximum rate (r), and threshold (k)^33^. Because s was not a useful descriptive parameter for the environmental samples^34^, only the parameters r and k were adopted in this study for characterizing the kinetic profiles of color development (Table 1). Kinetic analysis results showed that the utilization rates and capacity of carboxylic acids and phenolic compounds were quite high and relatively stable in BIO-amended rhizosphere soil along with the planting years (Table 1). Many studies reported the potential role of phenolic compounds and volatile (short-chain) fatty acids as allelopathic agents that were involved in the soil sickness^35^,^36^ of natural and managed ecosystems^37^. These autotoxins enhanced the cucumber root disease caused by *Fusarium oxysporum*^35^. Hence, the enhanced metabolic capacity for carboxylic acids and phenolic compounds after BIO application might decrease the autotoxic effects in continuous cropping systems and might contribute to plant disease resistance to pathogens. This finding was in accordance with some investigations that also suggested that autotoxicity could be overcome or alleviated by proper soil and plant residue management in addition to microbial degradation^36^. Moreover, a number of researches on the negative relation between the sugar contents of root exudates and plant resistance to *Fusarium* wilt or *Verticillium* wilt were documented^38^. Consistent with these observations, our results clearly showed that the enhanced metabolic capacity for carbohydrates in soils amended with BIO was also benefitable for the plant’s defense against Fusarium wilt. The PCA and kinetics analysis, both demonstrated that the lower carbon metabolism function was observed in CK rhizosphere soils along with the planting years, which indicated a degradation trend in a soil ecosystem^39^. In contrast, the stable general microbial metabolic potential was detected in fields applied by BIO. Therefore, BIO application could preserve the soil stability in view of the carbon metabolism functions.

The DGGE fingerprints were analyzed by UPGMA, and the results demonstrated that the community structure of soil culturable bacteria varied greatly in the newly reclaimed land under standard management (CK), whereas the community structure under BIO treatment was relatively stable and more resilient(Fig. 5b,d). In accordance with these results, Zhang *et al*.^40^ noted that a healthy and stable soil microstructure was essential to maintaining long-term continuous cropping and stable high crop yield. A previous study demonstrated that bacterial diversity promoted the community stability and functional resilience after perturbation^41^. Consistent with these observations, higher culturable bacteria richness and diversity were detected in the soil to which BIO was applied and lower richness and diversity indexes were observed with CK treatment, especially in the diseased plants (Supplementary Table S3). Collectively, bioorganic fertilizer that suppressed the banana wilt might be related to the resilient community structure and higher diversity of culturable bacteria in soil. This healthy application method likely maintained the healthy soil pattern that was suitable for continuous banana cropping.

Sequences of the dominant bands excised from the rhizosphere soil DGGE gels revealed that BIO application induced the enrichment of bands 1 to 8, which were affiliated with *Firmicutes*, *Proteobacteria* and *Actinobacteria* (Table 2). This finding was consistent with earlier report that these phyla were consistently associated with disease suppression^42^. Bands 1, 2, and 4 became more pronounced in BIO treatment, and they were identified as *Bacillus megaterium*, *Bacillus stratosphericus*, *and Rhizobium* sp., respectively. Bands 3, 5, 6, and 8 were unique and (or) constant under BIO treatment, and they were classified as *Pseudomonas fluorescens*, *Burkholderia* sp., *Actinobacterium* and *Brevibacillus formosu,* respectively. It is well known that many *Bacillus* and *Pseudomonas* species can contribute to plant growth and disease suppression in many ways, and they have been identified as the biocontrol agents of many pathogens^43^,^44^. Cao *et al*.^45^ reported that the endophytic *Streptomycete* (affiliated with actinobateria) was antagonistic to the banana Fusarium wilt pathogen. The genus *Burkholderia* was also reported to be a biocontrol agent with broad-spectrum antimicrobial activity against many pathogenic fungi, such as *Fusarium* sp., *Phytophthora capsici*, *R. solani* and others^46^. The Rhizobium strains are known to interact positively with plant roots by producing IAA^47^. Unfortunately, we failed to recover *Bacillus amyloliquefaciens* during sequencing of selected discriminating PCR-DGGE bands. We speculated that several possible reasons might be accountable for this problem. First, PCR-DGGE has some limitations per se in separation of relatively small DNA fragments and sensitivity of detection of rare community members^48^. The quantity of *Bacillus amyloliquefaciens* integrated with BIO, which was applied in soil is a very small percentage of soil microbes; therefore, the abundance of *Bacillus amyloliquefaciens* may be lower than the detection limit of PCR-DGGE. Second, only about 180bp were used for sequencing analysis of every excised band, the number of bases may not enough for exact identification among *Bacillus* species. Some other *Bacillus* species (*Bacillus megaterium* and *Bacillus stratosphericus*) were identified in BIO-amended rhizosphere soils, which showed closest affiliations with *Bacillus amyloliquefaciens*. Third, only partial bands were excised for sequencing analysis, the *Bacillus amyloliquefaciens* may exist in the other non-sequenced bands.

However, as a matter of fact, the mechanism of BIO applied to soil for suppressing the banana Fusarium wilt is not fully understood. In some cases, the special bacterium that we added into soil is hard to be detected, despite the disease incidence is low, the banana plants are robust and biological indexes of soil look good. Given that, we speculated that the potential mechanism of BIO to suppress the banana Fusarium wilt might be attributed to the case that strain NJN-6 accompanied by BIO application play a role to suppress growth and spore germination of FOC by the production of anti-fungal compounds^49^ in an initial short time. After that, the quantity of functional microbe may be decreased gradually and below the detection limit of current technology. In accordance with antagonistic coevolution^50^, sequential dominance of antagonistic phenotypes in disease suppressive soils may account for the decline of functional microbe that we added into soil. In short, combining all results in the present study, the main mechanism by which the BIO application suppressed the Fusarium wilt might be attributed to the fact that the BIO promoted the growth of some other microorganisms associated with the disease resistance and resulted in a general suppression consequently. Concerning the suppression associated microbes which were solely induced by BIO, we speculated that the differences in microflora of compost may attribute to this result. And the differences between composts BIO and CK is being sutdied. It’s also note worthing that microbes which was enriched in the BIO-treatment can be cultivated from the rhizosphere soil and seem to be potential microbial resources for suppressing the Fusarium wilt. With respect to those potential biocontrol agents, it will be the focus of our future work.

## Conclusions

In the present study, effective suppression of banana Fusarium wilt by the application of bioorganic fertilizer in newly reclaimed fields was realized in four consecutive years, and significant growth promotions in these fields were also achieved. The stabilized general bacterial metabolic potential, especially for carbohydrates, carboxylic acids and phenolic compounds and resilient community structure of culturable rhizobacteria with enriched beneficial microbes might be associated with soil suppression. This study revealed a new method to control Fusarium wilt of banana for long term banana cultivation.

## Materials and Methods

### Compost and bio-organic fertilizer

The pig manure compost used in this study was produced by Jiangsu Tianniang Co. Ltd., in Suzhou by composting pig manure at 30–70 °C for 25 days. It contained 45.5% organic matter, 1.3% N, 2.5% P_2_O_5_ and 0.9% K_2_O. The BIO was supplied by Jiangsu Xintiandi of Biological Fertilizer Engineering Center, Ltd. in Yixing, China. The BIO contained 40.2% organic matter, 2.6% N, 2.8% P_2_O_5_ and 1.2% K_2_O and was prepared by using a solid fermentation method according to Zhang *et al*.^12^. For the BIO production, the antagonistic bacterium *Bacillus amyloliquefaciens* NJN-6^14^ was inoculated into an organic mixture of amino acid fertilizer and pig manure composts at a ratio of 2:3 (w/w) for the solid fermentation process. The NJN-6 concentration in the bio-organic fertilizers was greater than 1 × 10^8^ CFU g^−1^ dry weight at the end of the fermentation period.

### Site description and experimental layout

The experimental site was located at Wen Qiu village Lin Gao County (19°812´N, 109°680´E), one of the most important banana production areas in Hainan Province, China. This region has a tropical monsoon climate with an average annual temperature and precipitation of 23 °C and 1418 mm, respectively. The soil in this field was characterized as Acric Ferralsols (according to World Reference Base for Soil Resources, 2014) with a pH of 4.98 (5:1water to soil ratio), and it contains 17.2 g·kg^−1^ organic matter, 0.91 g·kg^−1^ total N, 43.7 mg·kg^−1^ Olsen-P and 126 mg·kg^−1^ available K. Prior to this experiment started, this area had been cultivated with *Eucalyptus* for many years.

The field experiment was performed over four successive years from 2009 to 2012. The forestland was reclaimed for banana plantation in 2009 at first, and then two new areas that were adjacent to the previous one were reclaimed sequentially in 2010 and 2011. Hence, this experimental design provides the opportunity to collect soil samples to which BIO was applied from 1 to 3 years simultaneously. The field experiment included two treatments, (1) control treatment (CK), soil amended with pig manure and (2) bio-organic fertilizer treatment (BIO), soil amended with bio-organic fertilizer. Randomized complete block designs were applied in each field, and each treatment comprised three independent replicate plots. For the first year in different reclaimed fields, each plot with an area of 0.07 ha was planted with 170 banana tissue culture plantlets (*Musa acuminata* AAA Cavendish cv. Brazil). For the second and third years, robust suckers of banana trees were stayed for next seasonal cropping. Fertilization scheme were showed in Supplementary Table S1. Two-thirds of the compost and essential mineral fertilizers were applied as basic fertilizers before planting by using a rotary tiller. The remaining organic or bioorganic fertilizers and essential mineral fertilizers were applied as a top dressing during the banana bud stage. With the exception of fertilization, the other management techniques were same for all plots.

### Disease incidence and banana yield

After the tissue culture plantlets of banana transplanted into the field for 4 months, the plants infected with Foc based on visual observations of typical wilt symptoms^15^ were recorded up to 10 months post-transplantation when the disease incidence usually stabilized. Disease incidence (DI) was expressed as the percentage of diseased plants per total number of plants. Mature banana fruits from 30 banana trees were randomly selected and weighed for each treatment group, and the mean banana fruit yield of each banana tree was calculated.

### Soil sampling

Soil sampling was done in August 2012 before the bananas were harvested, as described by Bonilla *et al*.^51^. Based on visual observations of banana Fusarium wilt symptoms, the roots of healthy trees from the BIO-treated areas for one, two, and three years (BIO1H, BIO2H, BIO3H) were collected in turn; the roots of healthy (CK1H, CK2H, CK3H) and wilt trees (CK1W, CK2W, CK3W) in the control areas corresponding to different planting years were both collected. For each replicate, the fresh roots of five plants (healthy or wilt) at a distance of 10 cm from the trunk and 10–30 cm deep from the soil surface were mixed to obtain a composite rhizosphere soil as a subsample. Subsamples from three replicates per treatment were combined. All root samples were aseptically transferred to storage bags and maintained on ice prior to transport to the laboratory.

### Determining culturable rhizobacterial populations

The collected root samples were processed immediately after being brought to the lab according to Costa *et al*.^52^. In brief, the root tissues with their associated rhizosphere soil were cut into 1cm segments by using a sterile scalpel under aseptic conditions, and 10 g of these segments from different parts of the root in proportion was placed in 90 ml of sterile saline solution (0.85% NaCl) in a conical flask (250 ml). The mixtures in the flasks were homogenized by vigorous vortexing for 30 s and sonicated in an ultrasonic cleaner for 5 min. After that, the rhizosphere soil suspension was obtained by shaking the samples with glass beads (3 mm diameter) for 30 min on an orbital shaker at 170 rpm and room temperature (23 °C).

R2A medium, a non-selective medium recommended for the examination of total heterotrophic bacteria in soil^23^, was used to culture the heterotrophic population. Aliquots of 0.1 ml of each soil dilution ranging from 10^−3^ to 10^−6^ were spread on R2A medium plates and incubated for 7 days at 25 °C.

### Community level physiological profiles

Sole carbon source substrate utilization assays in Biolog Ecoplates (Biolog inc., Hayward, CA, USA) were used to generate community-level physiological profiles (CLPPs) by following the procedure of Garland^53^ with some modifications. In brief, a 1000-fold serial dilution of the rhizosphere soil suspension was made and 150 μl aliquots were added to each well in the microplates. An adjustment of the inoculum sizes was made at a soil: water ratio of approximately 1:10 (w:v) for all tested samples. Soil particles were not removed, nor allowed to settle, during any step in the extraction or inoculation^26^. The plates were then packed into polyethylene bags to reduce evaporation and were incubated in the dark at 25 °C. All the steps were performed in a sterile environment. Color development was measured at a wavelength of 590 nm every 24 h for up to 7 days by using an automated microtiter plate reader (Biolog Inc.) and the data were collected by using MICROLOG 4.01 software (Biolog Inc.).

Before data analysis, the optical densities (OD) for each well were corrected against the control well (no substrate; tetrazolium dye only) to eliminate background color generated from the substrates and soil suspension^54^. Because of detection limitations, the well optical density values that were negative or below 0.06 were set to zero according to Classen *et al*.^55^. As an indicator of general microbial activity, the average well color development (AWCD) was calculated for each plate at each reading time according to Gomez *et al*.^27^. To better interpret the large amount of Biolog data, a kinetic approach was adopted to enable comparisons of multiple time point readings. A non-linear regression model based on a modified logistic equation was used to fit the sigmoidal shape of the color development curve as follows:
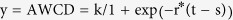
where k is the threshold or asymptote (the maximum absorbance in a well), r is the maximum rate of color development (as reflected by the slope of the curve), s is the time to the midpoint of the exponential portion of the curve (when y = K/2) and y is the absorbance at time t^33^,^34^. These parameters were estimated with the data analysis program Origin (version 8.0; Microcal Software, Inc., Northampton). This software uses the Levenberg-Marquardt algorithm and the simplex method for non-linear least-squares curve fitting, and it produced estimates of the model parameters and their standard errors along with an estimate for the goodness of fit (χ^2^). Principal component analysis (PCA, using Canoco for Windows 4.5) was then performed on sample data when the AWCD values were approximately equivalent to 0.8^56^.

### DNA extractions from isolates

Dilution plates that carried 50 to 500 colonies after 7 days of cultivation were selected for subsequent DNA extraction. Cultured cells were washed sequentially from 5 replicate plates into microcentrifuge tubes, and they were vortexed until the biomass from the plates was all homogenized. Bacterial genomic DNA was extracted from aliquots of each blended cell suspension (1.5 ml) by using the MoBio Microbial DNA extraction kit (MoBio Labs, Carlsbad, CA) according to the manufacturer’s instructions. The DNA was routinely checked for purity and molecular size by using conventional gel electrophoresis. Extracted genomic DNA was stored at −20 °C until use.

### Genetic diversity of culturable rhizobacteria

The microbial diversity of culturable soil populations was determined by PCR-DGGE. The variable V3 region of the 16S rRNA gene from the Eubacteria was amplified by using PRBA338F primer (5′-ACTCCTACGGGAGGCAGCAG-3′) with a GC clamp and PRUN518R primer (5′-ATTACCGCGGCTGCTGG-3′)^57^. The PCR amplification mixture consisted of 5 μl of 10 × PCR buffer (Mg^2+^ free), 4 μl of MgCl_2_ (25 mM), 4 μl of dNTP mixture (2.5 mM), 0.2 μM of each primer, 1 μl of template DNA, 0.2 μl of 5 units/μl Ex Taq polymerase (Takara, Dalian, China) and sterile water to a total volume of 50 μl. The PCR amplification was performed at 94 °C for 5 min, followed by 32 cycles of 94 °C for 30 s, 60 °C for 45 s and 72 °C for 45 s, and a final extension at 72 °C for 10 min. The PCR products were electrophoresed on 1.5% (wt/vol) agarose to verify their size and quality.

DGGE analyses were performed with a D-Code Universal Detection System (Bio-Rad Laboratories, Hercules, CA, USA) according to Muyzer *et al*.^58^. Twenty microliter PCR product samples with 5 μl of loading buffer dye were loaded onto an 8% (w/v) polyacrylamide gel (acrylamide: bisacrylamide, 37.5: 1) with denaturing gradients ranging from 40% to 60% [the 100% denaturant was 7 M urea plus 40% (v/v) deionized formamide]. Electrophoresis was initiated by a 10 min pre-running step at a voltage of 200 V and the samples were subsequently run in 1 × TAE buffer (40 mM Tris-acetate, 1 mM EDTA, pH 8.0) at 60 °C for 16 h at a constant voltage of 80 V. The PCR products run for DGGE fingerprinting were done in duplicates. After electrophoresis, the gel was visualized by silver staining and scanned by using a scanner (Epson perfection v33, Seiko Epson Corporation, Japan). Bands of interest were excised and sequenced, as described by Ellis *et al*.^23^.

The DGGE images were analyzed by Quantity One software (Version 4.6.3, Bio-Rad Laboratories) (*p* < 0.05) for band detection and intensity. Cluster analysis was performed with the UPGMA algorithm (an unweighted pair group method using arithmetic means) to study the general patterns of community similarity in Quantity One (Version 4.6.3, Bio-Rad). The relative intensity of a specific band was expressed as the ratio between the intensity of that band and the total intensity of all bands in that lane. The species richness was calculated as the number of bands per sample. The Shannon-Wiener diversity index (*H′*) and evenness were calculated by using the number of bands and peak intensities^59^.

### Statistical analysis

Statistical analysis was performed by using the IMBSPSS statistics Version 20 software program (IBM Corporation, New York, United States). For all data, one-way analysis of variance (ANOVA) followed by Fisher’s least significant difference (LSD) *post*-*hoc* test were used for multiple comparisons at a significance level of *P* < 0.05. Non-normal data were square-root or log transformed.

## Additional Information

**How to cite this article**: Fu, L. *et al*. Continous application of bioorganic fertilizer induced resilient culturable bacteria community associated with banana Fusarium wilt suppression. *Sci. Rep.* **6**, 27731; doi: 10.1038/srep27731 (2016).

## Supplementary Material

Supplementary Information

## Figures and Tables

**Table 1:** Values of kinetic parameters for functional group of carbon sources for different rhizosphere soil samples.

**Kinetic Parameter**	**Sample**^a^ **ID**	**Carbohydrates**	**Amino acids**	**Polymers**	**Amines**	**Carboxylic acids**	**Phenolic compounds**
k	BIO1H	1.908 (0.059)ab	1.876 (0.038)d	1.911 (0.028)c	1.736 (0.027)d	1.820 (0.034)bc	2.105 (0.034)a
	BIO2H	1.977 (0.048)a	2.179 (0.075)a	2.254 (0.035)a	2.149 (0.025)a	2.048 (0.055)a	2.192 (0.053)a
	BIO3H	1.788 (0.048)bc	1.945 (0.029)cd	1.865 (0.025)c	2.056 (0.031)b	1.873 (0.036)b	2.051 (0.022)ab
	CK1H	1.892 (0.028)ab	2.108 (0.047)ab	2.053 (0.032)b	1.939 (0.038)c	1.642 (0.070)de	1.880 (0.029)c
	CK2H	1.683 (0.049)de	1.425 (0.014)f	1.429 (0.046)e	1.524 (0.026)e	1.302 (0.048)f	1.176 (0.067)d
	CK3H	1.571 (0.039)e	1.375 (0.021)f	1.367 (0.054)e	1.454 (0.019)e	1.321 (0.048)f	1.101 (0.041)d
	CK1W	1.735 (0.031)cd	1.741 (0.046)e	1.631 (0.047)d	1.727 (0.026)d	1.513 (0.038)e	1.838 (0.034)c
	CK2W	1.843 (0.058)abc	2.059 (0.049)bc	1.966 (0.038)bc	1.781 (0.014)d	1.672 (0.066)cd	1.919 (0.069)bc
	CK3W	1.373 (0.021)f	1.028 (0.022)g	1.022 (0.033)f	1.177 (0.021)f	0.889 (0.038)g	1.151 (0.051)d
r	BIO1H	0.062 (0.011)b	0.063 (0.007)bc	0.061 (0.005)ab	0.096 (0.011)ab	0.044 (0.003)a	0.063 (0.005)ab
	BIO2H	0.077 (0.012)b	0.054 (0.008)c	0.041 (0.002)c	0.109 (0.011)a	0.046 (0.005)a	0.063 (0.006)ab
	BIO3H	0.154 (0.071)a	0.067 (0.006)bc	0.066 (0.005)a	0.076 (0.007)bc	0.065 (0.007)a	0.074 (0.005)a
	CK1H	0.119 (0.014)ab	0.064 (0.007)bc	0.058 (0.004)abc	0.099 (0.014)ab	0.056 (0.012)a	0.066 (0.005)ab
	CK2H	0.070 (0.014)b	0.070 (0.004)bc	0.072 (0.011)a	0.088 (0.01)abc	0.063 (0.013)a	0.055 (0.010)b
	CK3H	0.071 (0.011)b	0.082 (0.007)ab	0.043 (0.004)c	0.076 (0.005)bc	0.067 (0.014)a	0.062 (0.009)ab
	CK1W	0.105 (0.017)ab	0.059 (0.008)bc	0.064 (0.009)a	0.079 (0.007)bc	0.065 (0.009)a	0.062 (0.006)ab
	CK2W	0.082 (0.017)ab	0.054 (0.006)c	0.044 (0.003)bc	0.089 (0.005)abc	0.067 (0.011)a	0.052 (0.006)b
	CK3W	0.112 (0.018)ab	0.103 (0.015)a	0.055 (0.007)abc	0.067 (0.008)c	0.058 (0.014)a	0.061 (0.011)ab

The data shown are the means and standard errors (in parenthesis, n = 3), and different letters in the same colomn following the SE indicate significant differences among different rhizosphere soil samples in each kinetic parameter (*P* < 0.05, ANOVA).

^a^BIO1H, BIO2H, BIO3H refer to the healthy plants in fields amend with BIO for one year, two years and three years; CK1H, CK2H, CK3H and CK1W, CK2W, CK3W refer to the healthy plants and wilt plants in CK treated areas for one year, two years and three years respectively.

**Table 2:** Sequencing and taxonomic affiliation of the excised bands from the DGGE gels.

**DGGE Band NO.**	**Closest relative microorganisms (Accession number)**	**Similarity (%)**^a^	**Taxonomic group**
1	*Bacillus megaterium* (KC405251.1)	99	Firmicutes
2	*Bacillus stratosphericus* (JX438704.1)	100	Firmicutes
3	*Pseudomonas fluorescens* (HF678366.1)	100	Gammaproteobacteria
4	*Rhizobium* sp. (JX292656.1)	99	Firmicutes
5	*Burkholderia* sp. (JQ437431.1)	95	Gammaproteobacteria
6	*Actinobacterium* (EU723156.1)	98	Actinobacteria
7	*Acinetobacter baumannii* (HE651910.1)	100	Gammaproteobacteria
8	*Brevibacillus formosu* (KC495122.1)	100	Firmicutes
9	*Chryseobacterium gleum* (HE800565.1)	100	Bacteroidetes
10	*Flavobacterium* sp. (JX909005.1)	100	Bacteroidetes
11	*Chitinophaga* sp. (GQ281772.1)	100	Bacteroidetes
12	*Sinorhizobium meliloti* (KC460413.1)	99	Alphaproteobacteria
13	*Agrobacterium tumefacien* (KC355320.1)	100	Alphaproteobacteria
14	*Rhizobium huautlense* (KC355318.1)	99	Alphaproteobacteria
15	*Pseudoxanthomonas* sp. (KC170364.1)	99	Gammaproteobacteria
16	*Chitinophagaceae bacterium* (JN616363.1)	96	Bacteroidetes
17	*Pedobacter* sp. (JX909183.1)	99	Gammaproteobacteria
18	*Cronobacter sakazakii* (JX035786.1)	99	Bacteroidetes
19	*Novosphingobium* sp. (KC410867.1)	100	Bacteroidetes
20	*Sphingobium* sp. (JQ659689.1)	98	Bacteroidetes

^a^Sequences were aligned against the GenBank database with the BLAST search alignment tool.
