# Supplementary material for: Continous application of bioorganic fertilizer induced resilient culturable bacteria community associated with banana Fusarium wilt suppression
Source: Sci Rep. 2016 Jun 16;6:27731. doi: 10.1038/srep27731 (PMC4910074; doi:10.1038/srep27731)
Supplement: Supplementary Information [file srep27731-s1.pdf]

Title: Continous application of bioorganic fertilizer induced resilient culturable bacteria community associated with banana Fusarium wilt suppression

Authors: Lin Fu, Yunze Ruan, Chengyuan Tao, Rong Li, and Qirong Shen

Supplementary Table S1. Detailed fertilization scheme in field experiment from 2009 to 2012 (Hainan, China)

Treatment	Fertilizer	Fertilizing amount (kg ha^{-1} y^{-1})					
		Field I (reclaimed in 2009)			Field II(reclaimed in 2010)		Field III (reclaimed in 2011)
		2009-2010	2010-2011	2011-2012	2010-2011	2011-2012	2011-2012
BIO	Bio-organic fertilizer	12000	8000	8000	12000	8000	12000
	N	288	212	212	288	212	288
	P	110	89	89	110	89	110
	K	1156	814	814	1156	814	1156
CK	Pig manure	12000	8000	8000	12000	8000	12000
	N	445	316	316	445	316	445
	P	146	113	113	146	113	146
	K	1192	838	838	1192	838	1192

Supplementary Table S2. Carbon sources in the Biolog EcoPlate™

Biolog code	Substrate	Functional group
A2	β-Methyl-D-glucoside	Carbohydrates
A3	D-Galactonic acid γ-lactone	Carboxylic acids
A4	L-Arginine	Amino acids
B1	Pyruvic acid methyl ester	Carboxylic acids
B2	D-Xylose	Carbohydrates
B3	D-Galacturonic acid	Carboxylic acids
B4	L-Asparagine	Amino acids
C1	Tween 40	Polymers
C2	i-Erythriol	Carbohydrates
C3	2-Hydroxy benzoic acid	Phenolic compounds
C4	L-Phenylalanine	Amino acids
D1	Tween 80	Polymers
D2	D-Mannitol	Carbohydrates
D3	4-Hydroxy benzoic acid	Phenolic compounds
D4	L-Serine	Amino acids
E1	α-Cyclodextrin	Polymers
E2	N-Aceryl-D-glucosamine	Carbohydrates
E3	γ-Hydroxybutyric acid	Carboxylic acids
E4	L-Threonine	Amino acids
F1	Glycogen	Polymers

F2	D-Glucosaminic acid	Carboxylic acids
F3	Itaconic acid	Carboxylic acids
F4	Glycyl-L-glutamic acid	Amino acids
G1	D-Cellobiose	Carbohydrates
G2	Glucose-1-phosphate	Carbohydrates
G3	α-Ketobutyric acid	Carboxylic acids
G4	Phenylethyl-amine	Amines
H1	α-D-Lactose	Carbohydrates
H2	D,L-α-Glycerol phosphate	Carbohydrates
H3	D-Malic acid	Carboxylic acids
H4	Putrescine	Amines

Supplementary Table S3. Richness (R), Shannon-Wiener diversity indices (H') and evenness (E) for culturable bacteria communities analyzed by PCR-DGGE of different samples

Sample[a] ID	Richness (R)	Diversity (H')	Evenness (E)
(a)[b] BIO1H	22(0)	2.80(0.01)	0.91(0.01)
BIO2H	18(0)	2.60(0.01)	0.90(0.01)
BIO3H	19(0)	2.81(0.03)	0.93(0.01)
CK1H	17(0)	2.52(0.01)	0.89(0.01)
CK2H	16(0)	2.49(0.02)	0.90(0.01)
CK3H	15(0)	2.57(0.01)	0.95(0.02)
(b)[c] BIO1H	22(0)	2.83(0.01)	0.94(0.01)
BIO2H	18(0)	2.51(0.01)	0.95(0.02)
BIO3H	18(0)	2.74(0.01)	0.97(0.01)
CK1W	12(0)	2.30(0.02)	0.92(0.01)
CK2W	12(0)	2.36(0.01)	0.95(0.01)
CK3W	12(0)	2.21(0.01)	0.92(0.01)

The data shown are the means and standard deviation (in parenthesis), n=2.

[a] BIO1H, BIO2H, BIO3H refer to the healthy plants in fields amend with BIO for one year, two years and three years; CK1H, CK2H, CK3H and CK1W, CK2W, CK3W refer to the healthy plants and wilt plants in CK treated area for one year, two years and three years respectively.

[b] The indexes of samples in Fig. 5a.

[c] The indexes of samples in Fig. 5c.
